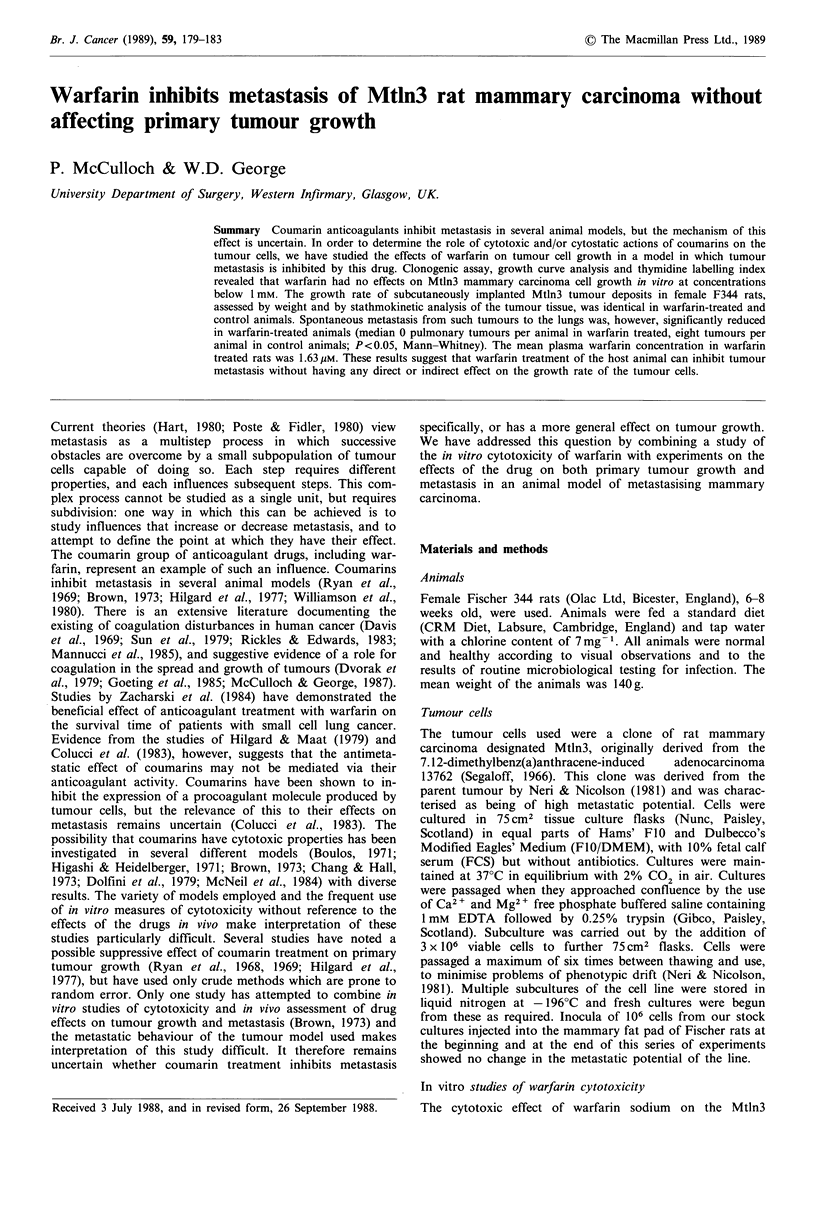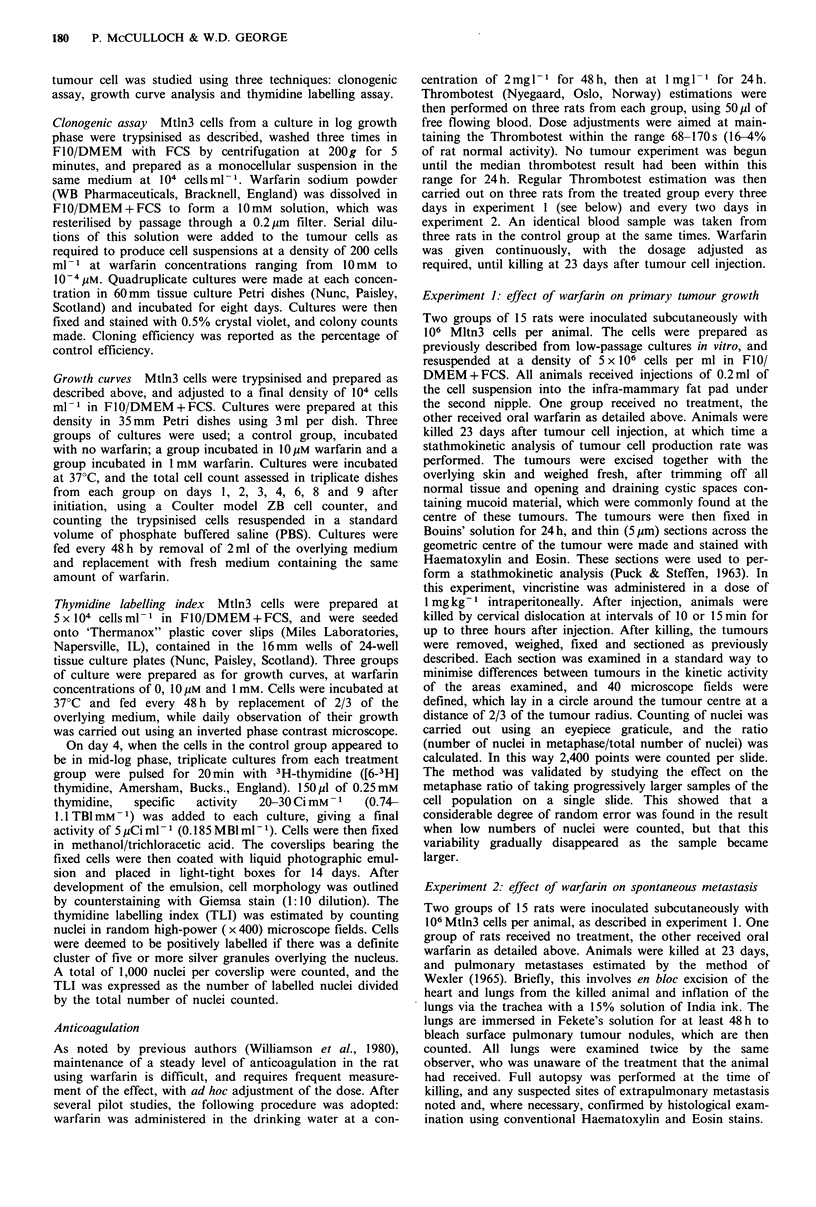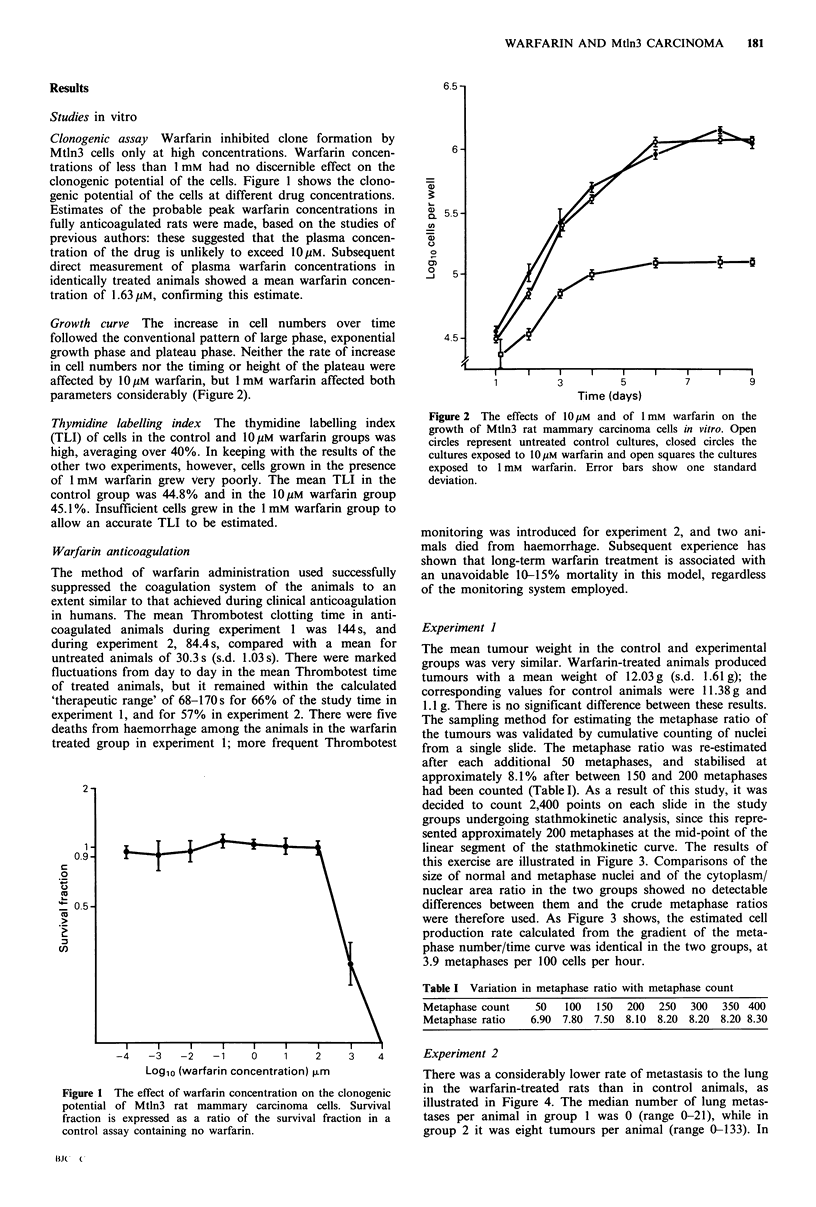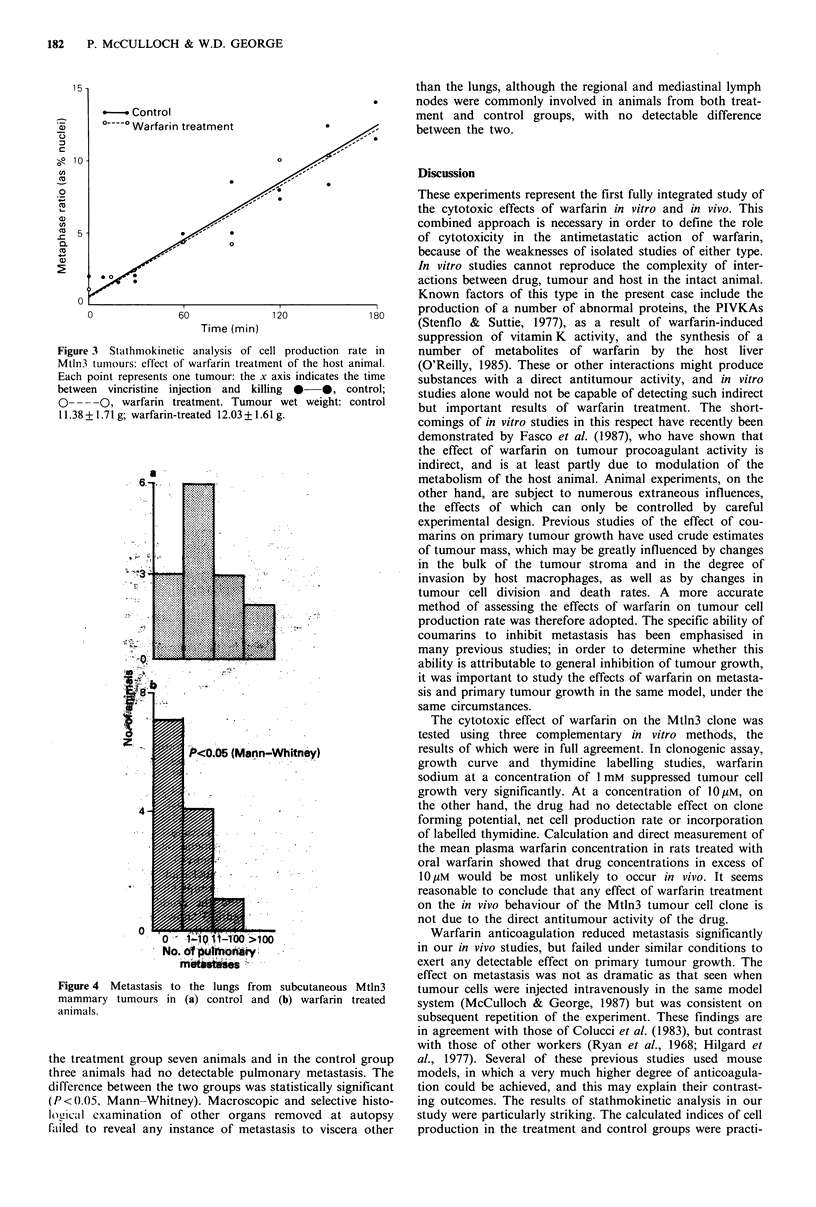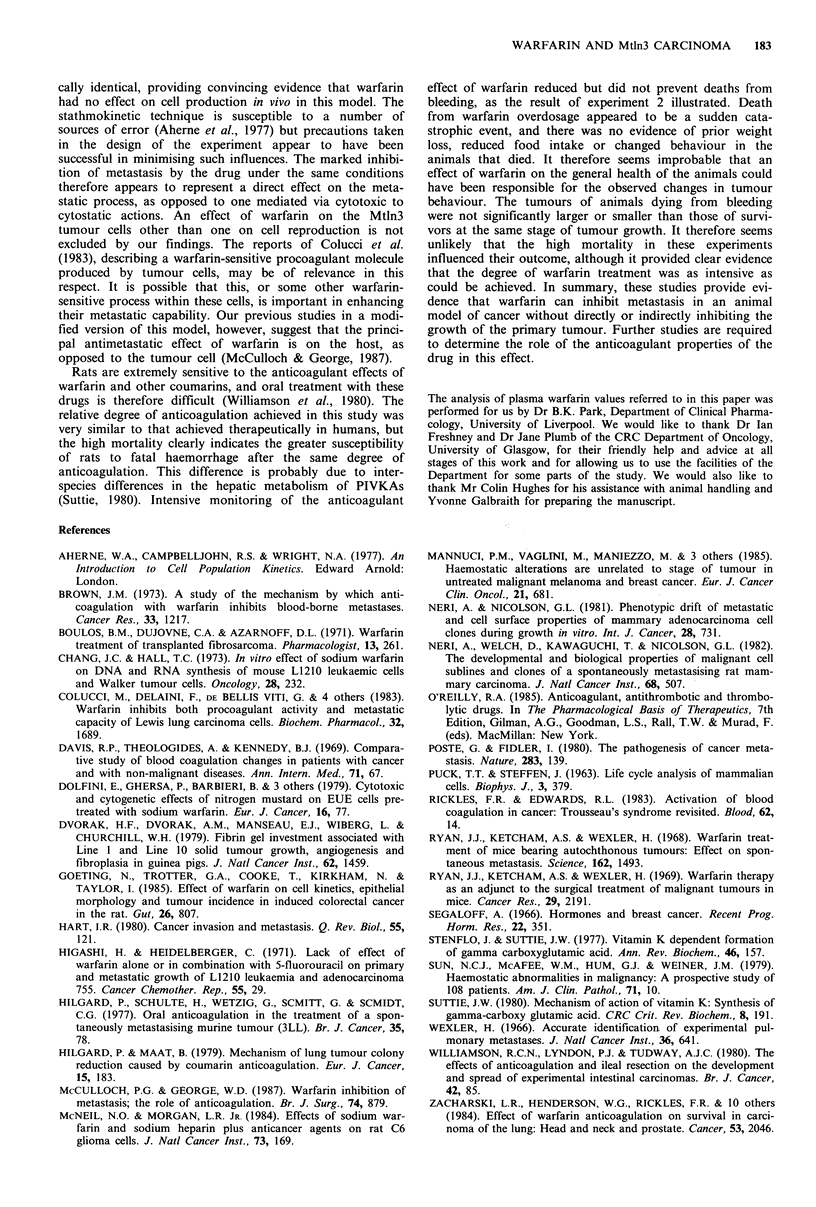# Warfarin inhibits metastasis of Mtln3 rat mammary carcinoma without affecting primary tumour growth.

**DOI:** 10.1038/bjc.1989.37

**Published:** 1989-02

**Authors:** P. McCulloch, W. D. George

**Affiliations:** University Department of Surgery, Western Infirmary, Glasgow, UK.

## Abstract

Coumarin anticoagulants inhibit metastasis in several animal models, but the mechanism of this effect is uncertain. In order to determine the role of cytotoxic and/or cytostatic actions of coumarins on the tumour cells, we have studied the effects of warfarin on tumour cell growth in a model in which tumour metastasis is inhibited by this drug. Clonogenic assay, growth curve analysis and thymidine labelling index revealed that warfarin had no effects on Mtln3 mammary carcinoma cell growth in vitro at concentrations below 1 mM. The growth rate of subcutaneously implanted Mtln3 tumour deposits in female F344 rats, assessed by weight and by stathmokinetic analysis of the tumour tissue, was identical in warfarin-treated and control animals. Spontaneous metastasis from such tumours to the lungs was, however, significantly reduced in warfarin-treated animals (median 0 pulmonary tumours per animal in warfarin treated, eight tumours per animal in control animals; P less than 0.05, Mann-Whitney). The mean plasma warfarin concentration in warfarin treated rats was 1.63 microM. These results suggest that warfarin treatment of the host animal can inhibit tumour metastasis without having any direct or indirect effect on the growth rate of the tumour cells.


*Br. J. Cancer* (1989), **59**, 179–183 © The Macmillan Press Ltd., 1989

### Warfarin inhibits metastasis of Mtln3 rat mammary carcinoma without affecting primary tumour growth

P. McCulloch & W.D. George

*University Department of Surgery, Western Infirmary, Glasgow, UK.*

**Summary** Coumarin anticoagulants inhibit metastasis in several animal models, but the mechanism of this effect is uncertain. In order to determine the role of cytotoxic and/or cytostatic actions of coumarins on the tumour cells, we have studied the effects of warfarin on tumour cell growth in a model in which tumour metastasis is inhibited by this drug. Clonogenic assay, growth curve analysis and thymidine labelling index revealed that warfarin had no effects on Mtln3 mammary carcinoma cell growth *in vitro* at concentrations below 1 mM. The growth rate of subcutaneously implanted Mtln3 tumour deposits in female F344 rats, assessed by weight and by stathmokinetic analysis of the tumour tissue, was identical in warfarin-treated and control animals. Spontaneous metastasis from such tumours to the lungs was, however, significantly reduced in warfarin-treated animals (median 0 pulmonary tumours per animal in warfarin treated, eight tumours per animal in control animals; $P<0.05$, Mann–Whitney). The mean plasma warfarin concentration in warfarin treated rats was 1.63 $\mu$M. These results suggest that warfarin treatment of the host animal can inhibit tumour metastasis without having any direct or indirect effect on the growth rate of the tumour cells.

Current theories (Hart, 1980; Poste & Fidler, 1980) view metastasis as a multistep process in which successive obstacles are overcome by a small subpopulation of tumour cells capable of doing so. Each step requires different properties, and each influences subsequent steps. This complex process cannot be studied as a single unit, but requires subdivision: one way in which this can be achieved is to study influences that increase or decrease metastasis, and to attempt to define the point at which they have their effect. The coumarin group of anticoagulant drugs, including warfarin, represent an example of such an influence. Coumarins inhibit metastasis in several animal models (Ryan *et al.*, 1969; Brown, 1973; Hilgard *et al.*, 1977; Williamson *et al.*, 1980). There is an extensive literature documenting the existing of coagulation disturbances in human cancer (Davis *et al.*, 1969; Sun *et al.*, 1979; Rickles & Edwards, 1983; Mannucci *et al.*, 1985), and suggestive evidence of a role for coagulation in the spread and growth of tumours (Dvorak *et al.*, 1979; Goeting *et al.*, 1985; McCulloch & George, 1987). Studies by Zacharski *et al.* (1984) have demonstrated the beneficial effect of anticoagulant treatment with warfarin on the survival time of patients with small cell lung cancer. Evidence from the studies of Hilgard & Maat (1979) and Colucci *et al.* (1983), however, suggests that the antimetastatic effect of coumarins may not be mediated via their anticoagulant activity. Coumarins have been shown to inhibit the expression of a procoagulant molecule produced by tumour cells, but the relevance of this to their effects on metastasis remains uncertain (Colucci *et al.*, 1983). The possibility that coumarins have cytotoxic properties has been investigated in several different models (Boulos, 1971; Higashi & Heidelberger, 1971; Brown, 1973; Chang & Hall, 1973; Dolfini *et al.*, 1979; McNeil *et al.*, 1984) with diverse results. The variety of models employed and the frequent use of *in vitro* measures of cytotoxicity without reference to the effects of the drugs *in vivo* make interpretation of these studies particularly difficult. Several studies have noted a possible suppressive effect of coumarin treatment on primary tumour growth (Ryan *et al.*, 1968, 1969; Hilgard *et al.*, 1977), but have used only crude methods which are prone to random error. Only one study has attempted to combine *in vitro* studies of cytotoxicity and *in vivo* assessment of drug effects on tumour growth and metastasis (Brown, 1973) and the metastatic behaviour of the tumour model used makes interpretation of this study difficult. It therefore remains uncertain whether coumarin treatment inhibits metastasis specifically, or has a more general effect on tumour growth. We have addressed this question by combining a study of the *in vitro* cytotoxicity of warfarin with experiments on the effects of the drug on both primary tumour growth and metastasis in an animal model of metastasising mammary carcinoma.

Received 3 July 1988, and in revised form, 26 September 1988.

### Materials and methods

### *Animals*

Female Fischer 344 rats (Olac Ltd, Bicester, England), 6–8 weeks old, were used. Animals were fed a standard diet (CRM Diet, Labsure, Cambridge, England) and tap water with a chlorine content of 7 mg$^{-1}$. All animals were normal and healthy according to visual observations and to the results of routine microbiological testing for infection. The mean weight of the animals was 140 g.

### *Tumour cells*

The tumour cells used were a clone of rat mammary carcinoma designated Mtln3, originally derived from the 7.12-dimethylbenz(a)anthracene-induced adenocarcinoma 13762 (Segaloff, 1966). This clone was derived from the parent tumour by Neri & Nicolson (1981) and was characterised as being of high metastatic potential. Cells were cultured in 75 cm$^2$ tissue culture flasks (Nunc, Paisley, Scotland) in equal parts of Hams' F10 and Dulbecco's Modified Eagles' Medium (F10/DMEM), with 10% fetal calf serum (FCS) but without antibiotics. Cultures were maintained at 37°C in equilibrium with 2% $CO_2$ in air. Cultures were passaged when they approached confluence by the use of $Ca^{2+}$ and $Mg^{2+}$ free phosphate buffered saline containing 1 mM EDTA followed by 0.25% trypsin (Gibco, Paisley, Scotland). Subculture was carried out by the addition of $3\times10^6$ viable cells to further 75 cm$^2$ flasks. Cells were passaged a maximum of six times between thawing and use, to minimise problems of phenotypic drift (Neri & Nicolson, 1981). Multiple subcultures of the cell line were stored in liquid nitrogen at −196°C and fresh cultures were begun from these as required. Inocula of $10^6$ cells from our stock cultures injected into the mammary fat pad of Fischer rats at the beginning and at the end of this series of experiments showed no change in the metastatic potential of the line.

### In vitro *studies of warfarin cytotoxicity*

The cytotoxic effect of warfarin sodium on the Mtln3

tumour cell was studied using three techniques: clonogenic assay, growth curve analysis and thymidine labelling assay.

*Clonogenic assay* Mtln3 cells from a culture in log growth phase were trypsinised as described, washed three times in F10/DMEM with FCS by centrifugation at 200*g* for 5 minutes, and prepared as a monocellular suspension in the same medium at $10^4$ cells $ml^{-1}$. Warfarin sodium powder (WB Pharmaceuticals, Bracknell, England) was dissolved in F10/DMEM + FCS to form a 10 mM solution, which was resterilised by passage through a 0.2 μm filter. Serial dilutions of this solution were added to the tumour cells as required to produce cell suspensions at a density of 200 cells $ml^{-1}$ at warfarin concentrations ranging from 10 mM to $10^{-4}$ μM. Quadruplicate cultures were made at each concentration in 60 mm tissue culture Petri dishes (Nunc, Paisley, Scotland) and incubated for eight days. Cultures were then fixed and stained with 0.5% crystal violet, and colony counts made. Cloning efficiency was reported as the percentage of control efficiency.

*Growth curves* Mtln3 cells were trypsinised and prepared as described above, and adjusted to a final density of $10^4$ cells $ml^{-1}$ in F10/DMEM + FCS. Cultures were prepared at this density in 35 mm Petri dishes using 3 ml per dish. Three groups of cultures were used; a control group, incubated with no warfarin; a group incubated in 10 μM warfarin and a group incubated in 1 mM warfarin. Cultures were incubated at 37°C, and the total cell count assessed in triplicate dishes from each group on days 1, 2, 3, 4, 6, 8 and 9 after initiation, using a Coulter model ZB cell counter, and counting the trypsinised cells resuspended in a standard volume of phosphate buffered saline (PBS). Cultures were fed every 48 h by removal of 2 ml of the overlying medium and replacement with fresh medium containing the same amount of warfarin.

*Thymidine labelling index* Mtln3 cells were prepared at $5 \times 10^4$ cells $ml^{-1}$ in F10/DMEM + FCS, and were seeded onto 'Thermanox" plastic cover slips (Miles Laboratories, Napersville, IL), contained in the 16 mm wells of 24-well tissue culture plates (Nunc, Paisley, Scotland). Three groups of culture were prepared as for growth curves, at warfarin concentrations of 0, 10 μM and 1 mM. Cells were incubated at 37°C and fed every 48 h by replacement of 2/3 of the overlying medium, while daily observation of their growth was carried out using an inverted phase contrast microscope.

On day 4, when the cells in the control group appeared to be in mid-log phase, triplicate cultures from each treatment group were pulsed for 20 min with $^3$H-thymidine ([6-$^3$H] thymidine, Amersham, Bucks., England). 150 μl of 0.25 mM thymidine, specific activity 20–30 Ci $mM^{-1}$ (0.74–1.1 TBl $mM^{-1}$) was added to each culture, giving a final activity of 5 μCi $ml^{-1}$ (0.185 MBl $ml^{-1}$). Cells were then fixed in methanol/trichloracetic acid. The coverslips bearing the fixed cells were then coated with liquid photographic emulsion and placed in light-tight boxes for 14 days. After development of the emulsion, cell morphology was outlined by counterstaining with Giemsa stain (1:10 dilution). The thymidine labelling index (TLI) was estimated by counting nuclei in random high-power ( × 400) microscope fields. Cells were deemed to be positively labelled if there was a definite cluster of five or more silver granules overlying the nucleus. A total of 1,000 nuclei per coverslip were counted, and the TLI was expressed as the number of labelled nuclei divided by the total number of nuclei counted.

*Anticoagulation*

As noted by previous authors (Williamson *et al.*, 1980), maintenance of a steady level of anticoagulation in the rat using warfarin is difficult, and requires frequent measurement of the effect, with *ad hoc* adjustment of the dose. After several pilot studies, the following procedure was adopted: warfarin was administered in the drinking water at a concentration of 2 mg $l^{-1}$ for 48 h, then at 1 mg $l^{-1}$ for 24 h. Thrombotest (Nyegaard, Oslo, Norway) estimations were then performed on three rats from each group, using 50 μl of free flowing blood. Dose adjustments were aimed at maintaining the Thrombotest within the range 68–170 s (16–4% of rat normal activity). No tumour experiment was begun until the median thrombotest result had been within this range for 24 h. Regular Thrombotest estimation was then carried out on three rats from the treated group every three days in experiment 1 (see below) and every two days in experiment 2. An identical blood sample was taken from three rats in the control group at the same times. Warfarin was given continuously, with the dosage adjusted as required, until killing at 23 days after tumour cell injection.

*Experiment 1: effect of warfarin on primary tumour growth*

Two groups of 15 rats were inoculated subcutaneously with $10^6$ Mltn3 cells per animal. The cells were prepared as previously described from low-passage cultures *in vitro*, and resuspended at a density of $5 \times 10^6$ cells per ml in F10/DMEM + FCS. All animals received injections of 0.2 ml of the cell suspension into the infra-mammary fat pad under the second nipple. One group received no treatment, the other received oral warfarin as detailed above. Animals were killed 23 days after tumour cell injection, at which time a stathmokinetic analysis of tumour cell production rate was performed. The tumours were excised together with the overlying skin and weighed fresh, after trimming off all normal tissue and opening and draining cystic spaces containing mucoid material, which were commonly found at the centre of these tumours. The tumours were then fixed in Bouins' solution for 24 h, and thin (5 μm) sections across the geometric centre of the tumour were made and stained with Haematoxylin and Eosin. These sections were used to perform a stathmokinetic analysis (Puck & Steffen, 1963). In this experiment, vincristine was administered in a dose of 1 mg $kg^{-1}$ intraperitoneally. After injection, animals were killed by cervical dislocation at intervals of 10 or 15 min for up to three hours after injection. After killing, the tumours were removed, weighed, fixed and sectioned as previously described. Each section was examined in a standard way to minimise differences between tumours in the kinetic activity of the areas examined, and 40 microscope fields were defined, which lay in a circle around the tumour centre at a distance of 2/3 of the tumour radius. Counting of nuclei was carried out using an eyepiece graticule, and the ratio (number of nuclei in metaphase/total number of nuclei) was calculated. In this way 2,400 points were counted per slide. The method was validated by studying the effect on the metaphase ratio of taking progressively larger samples of the cell population on a single slide. This showed that a considerable degree of random error was found in the result when low numbers of nuclei were counted, but that this variability gradually disappeared as the sample became larger.

*Experiment 2: effect of warfarin on spontaneous metastasis*

Two groups of 15 rats were inoculated subcutaneously with $10^6$ Mltn3 cells per animal, as described in experiment 1. One group of rats received no treatment, the other received oral warfarin as detailed above. Animals were killed at 23 days, and pulmonary metastases estimated by the method of Wexler (1965). Briefly, this involves *en bloc* excision of the heart and lungs from the killed animal and inflation of the lungs via the trachea with a 15% solution of India ink. The lungs are immersed in Fekete's solution for at least 48 h to bleach surface pulmonary tumour nodules, which are then counted. All lungs were examined twice by the same observer, who was unaware of the treatment that the animal had received. Full autopsy was performed at the time of killing, and any suspected sites of extrapulmonary metastasis noted and, where necessary, confirmed by histological examination using conventional Haematoxylin and Eosin stains.

### Results

### *Studies* in vitro

*Clonogenic assay* Warfarin inhibited clone formation by Mtln3 cells only at high concentrations. Warfarin concentrations of less than 1 mM had no discernible effect on the clonogenic potential of the cells. Figure 1 shows the clonogenic potential of the cells at different drug concentrations. Estimates of the probable peak warfarin concentrations in fully anticoagulated rats were made, based on the studies of previous authors: these suggested that the plasma concentration of the drug is unlikely to exceed 10 $\mu$M. Subsequent direct measurement of plasma warfarin concentrations in identically treated animals showed a mean warfarin concentration of 1.63 $\mu$M, confirming this estimate.

*Growth curve* The increase in cell numbers over time followed the conventional pattern of large phase, exponential growth phase and plateau phase. Neither the rate of increase in cell numbers nor the timing or height of the plateau were affected by 10 $\mu$M warfarin, but 1 mM warfarin affected both parameters considerably (Figure 2).

*Thymidine labelling index* The thymidine labelling index (TLI) of cells in the control and 10 $\mu$M warfarin groups was high, averaging over 40%. In keeping with the results of the other two experiments, however, cells grown in the presence of 1 mM warfarin grew very poorly. The mean TLI in the control group was 44.8% and in the 10 $\mu$M warfarin group 45.1%. Insufficient cells grew in the 1 mM warfarin group to allow an accurate TLI to be estimated.

### *Warfarin anticoagulation*

The method of warfarin administration used successfully suppressed the coagulation system of the animals to an extent similar to that achieved during clinical anticoagulation in humans. The mean Thrombotest clotting time in anticoagulated animals during experiment 1 was 144 s, and during experiment 2, 84.4 s, compared with a mean for untreated animals of 30.3 s (s.d. 1.03 s). There were marked fluctuations from day to day in the mean Thrombotest time of treated animals, but it remained within the calculated 'therapeutic range' of 68–170 s for 66% of the study time in experiment 1, and for 57% in experiment 2. There were five deaths from haemorrhage among the animals in the warfarin treated group in experiment 1; more frequent Thrombotest monitoring was introduced for experiment 2, and two animals died from haemorrhage. Subsequent experience has shown that long-term warfarin treatment is associated with an unavoidable 10–15% mortality in this model, regardless of the monitoring system employed.

**Figure 1** The effect of warfarin concentration on the clonogenic potential of Mtln3 rat mammary carcinoma cells. Survival fraction is expressed as a ratio of the survival fraction in a control assay containing no warfarin.

**Figure 2** The effects of 10 $\mu$M and of 1 mM warfarin on the growth of Mtln3 rat mammary carcinoma cells *in vitro*. Open circles represent untreated control cultures, closed circles the cultures exposed to 10 $\mu$M warfarin and open squares the cultures exposed to 1 mM warfarin. Error bars show one standard deviation.

### *Experiment 1*

The mean tumour weight in the control and experimental groups was very similar. Warfarin-treated animals produced tumours with a mean weight of 12.03 g (s.d. 1.61 g); the corresponding values for control animals were 11.38 g and 1.1 g. There is no significant difference between these results. The sampling method for estimating the metaphase ratio of the tumours was validated by cumulative counting of nuclei from a single slide. The metaphase ratio was re-estimated after each additional 50 metaphases, and stabilised at approximately 8.1% after between 150 and 200 metaphases had been counted (Table I). As a result of this study, it was decided to count 2,400 points on each slide in the study groups undergoing stathmokinetic analysis, since this represented approximately 200 metaphases at the mid-point of the linear segment of the stathmokinetic curve. The results of this exercise are illustrated in Figure 3. Comparisons of the size of normal and metaphase nuclei and of the cytoplasm/nuclear area ratio in the two groups showed no detectable differences between them and the crude metaphase ratios were therefore used. As Figure 3 shows, the estimated cell production rate calculated from the gradient of the metaphase number/time curve was identical in the two groups, at 3.9 metaphases per 100 cells per hour.

**Table I** Variation in metaphase ratio with metaphase count

| Metaphase count | 50 | 100 | 150 | 200 | 250 | 300 | 350 | 400 |
|---|---|---|---|---|---|---|---|---|
| Metaphase ratio | 6.90 | 7.80 | 7.50 | 8.10 | 8.20 | 8.20 | 8.20 | 8.30 |

### *Experiment 2*

There was a considerably lower rate of metastasis to the lung in the warfarin-treated rats than in control animals, as illustrated in Figure 4. The median number of lung metastases per animal in group 1 was 0 (range 0–21), while in group 2 it was eight tumours per animal (range 0–133). In

**Figure 3** Stathmokinetic analysis of cell production rate in Mtln3 tumours: effect of warfarin treatment of the host animal. Each point represents one tumour: the $x$ axis indicates the time between vincristine injection and killing ●——●, control; ○----○, warfarin treatment. Tumour wet weight: control 11.38±1.71 g; warfarin-treated 12.03±1.61 g.

**Figure 4** Metastasis to the lungs from subcutaneous Mtln3 mammary tumours in (**a**) control and (**b**) warfarin treated animals.

the treatment group seven animals and in the control group three animals had no detectable pulmonary metastasis. The difference between the two groups was statistically significant ($P<0.05$, Mann–Whitney). Macroscopic and selective histological examination of other organs removed at autopsy failed to reveal any instance of metastasis to viscera other than the lungs, although the regional and mediastinal lymph nodes were commonly involved in animals from both treatment and control groups, with no detectable difference between the two.

### Discussion

These experiments represent the first fully integrated study of the cytotoxic effects of warfarin *in vitro* and *in vivo*. This combined approach is necessary in order to define the role of cytotoxicity in the antimetastatic action of warfarin, because of the weaknesses of isolated studies of either type. *In vitro* studies cannot reproduce the complexity of interactions between drug, tumour and host in the intact animal. Known factors of this type in the present case include the production of a number of abnormal proteins, the PIVKAs (Stenflo & Suttie, 1977), as a result of warfarin-induced suppression of vitamin K activity, and the synthesis of a number of metabolites of warfarin by the host liver (O'Reilly, 1985). These or other interactions might produce substances with a direct antitumour activity, and *in vitro* studies alone would not be capable of detecting such indirect but important results of warfarin treatment. The shortcomings of *in vitro* studies in this respect have recently been demonstrated by Fasco *et al.* (1987), who have shown that the effect of warfarin on tumour procoagulant activity is indirect, and is at least partly due to modulation of the metabolism of the host animal. Animal experiments, on the other hand, are subject to numerous extraneous influences, the effects of which can only be controlled by careful experimental design. Previous studies of the effect of coumarins on primary tumour growth have used crude estimates of tumour mass, which may be greatly influenced by changes in the bulk of the tumour stroma and in the degree of invasion by host macrophages, as well as by changes in tumour cell division and death rates. A more accurate method of assessing the effects of warfarin on tumour cell production rate was therefore adopted. The specific ability of coumarins to inhibit metastasis has been emphasised in many previous studies; in order to determine whether this ability is attributable to general inhibition of tumour growth, it was important to study the effects of warfarin on metastasis and primary tumour growth in the same model, under the same circumstances.

The cytotoxic effect of warfarin on the Mtln3 clone was tested using three complementary *in vitro* methods, the results of which were in full agreement. In clonogenic assay, growth curve and thymidine labelling studies, warfarin sodium at a concentration of 1 mM suppressed tumour cell growth very significantly. At a concentration of 10 $\mu$M, on the other hand, the drug had no detectable effect on clone forming potential, net cell production rate or incorporation of labelled thymidine. Calculation and direct measurement of the mean plasma warfarin concentration in rats treated with oral warfarin showed that drug concentrations in excess of 10 $\mu$M would be most unlikely to occur *in vivo*. It seems reasonable to conclude that any effect of warfarin treatment on the *in vivo* behaviour of the Mtln3 tumour cell clone is not due to the direct antitumour activity of the drug.

Warfarin anticoagulation reduced metastasis significantly in our *in vivo* studies, but failed under similar conditions to exert any detectable effect on primary tumour growth. The effect on metastasis was not as dramatic as that seen when tumour cells were injected intravenously in the same model system (McCulloch & George, 1987) but was consistent on subsequent repetition of the experiment. These findings are in agreement with those of Colucci *et al.* (1983), but contrast with those of other workers (Ryan *et al.*, 1968; Hilgard *et al.*, 1977). Several of these previous studies used mouse models, in which a very much higher degree of anticoagulation could be achieved, and this may explain their contrasting outcomes. The results of stathmokinetic analysis in our study were particularly striking. The calculated indices of cell production in the treatment and control groups were practically identical, providing convincing evidence that warfarin had no effect on cell production *in vivo* in this model. The stathmokinetic technique is susceptible to a number of sources of error (Aherne *et al.*, 1977) but precautions taken in the design of the experiment appear to have been successful in minimising such influences. The marked inhibition of metastasis by the drug under the same conditions therefore appears to represent a direct effect on the metastatic process, as opposed to one mediated via cytotoxic to cytostatic actions. An effect of warfarin on the Mtln3 tumour cells other than one on cell reproduction is not excluded by our findings. The reports of Colucci *et al.* (1983), describing a warfarin-sensitive procoagulant molecule produced by tumour cells, may be of relevance in this respect. It is possible that this, or some other warfarin-sensitive process within these cells, is important in enhancing their metastatic capability. Our previous studies in a modified version of this model, however, suggest that the principal antimetastatic effect of warfarin is on the host, as opposed to the tumour cell (McCulloch & George, 1987).

Rats are extremely sensitive to the anticoagulant effects of warfarin and other coumarins, and oral treatment with these drugs is therefore difficult (Williamson *et al.*, 1980). The relative degree of anticoagulation achieved in this study was very similar to that achieved therapeutically in humans, but the high mortality clearly indicates the greater susceptibility of rats to fatal haemorrhage after the same degree of anticoagulation. This difference is probably due to interspecies differences in the hepatic metabolism of PIVKAs (Suttie, 1980). Intensive monitoring of the anticoagulant effect of warfarin reduced but did not prevent deaths from bleeding, as the result of experiment 2 illustrated. Death from warfarin overdosage appeared to be a sudden catastrophic event, and there was no evidence of prior weight loss, reduced food intake or changed behaviour in the animals that died. It therefore seems improbable that an effect of warfarin on the general health of the animals could have been responsible for the observed changes in tumour behaviour. The tumours of animals dying from bleeding were not significantly larger or smaller than those of survivors at the same stage of tumour growth. It therefore seems unlikely that the high mortality in these experiments influenced their outcome, although it provided clear evidence that the degree of warfarin treatment was as intensive as could be achieved. In summary, these studies provide evidence that warfarin can inhibit metastasis in an animal model of cancer without directly or indirectly inhibiting the growth of the primary tumour. Further studies are required to determine the role of the anticoagulant properties of the drug in this effect.

The analysis of plasma warfarin values referred to in this paper was performed for us by Dr B.K. Park, Department of Clinical Pharmacology, University of Liverpool. We would like to thank Dr Ian Freshney and Dr Jane Plumb of the CRC Department of Oncology, University of Glasgow, for their friendly help and advice at all stages of this work and for allowing us to use the facilities of the Department for some parts of the study. We would also like to thank Mr Colin Hughes for his assistance with animal handling and Yvonne Galbraith for preparing the manuscript.

### References

AHERNE, W.A., CAMPBELLJOHN, R.S. & WRIGHT, N.A. (1977). *An Introduction to Cell Population Kinetics*. Edward Arnold: London.

BROWN, J.M. (1973). A study of the mechanism by which anticoagulation with warfarin inhibits blood-borne metastases. *Cancer Res.*, **33**, 1217.

BOULOS, B.M., DUJOVNE, C.A. & AZARNOFF, D.L. (1971). Warfarin treatment of transplanted fibrosarcoma. *Pharmacologist*, **13**, 261.

CHANG, J.C. & HALL, T.C. (1973). *In vitro* effect of sodium warfarin on DNA and RNA synthesis of mouse L1210 leukaemic cells and Walker tumour cells. *Oncology*, **28**, 232.

COLUCCI, M., DELAINI, F., DE BELLIS VITI, G. & 4 others (1983). Warfarin inhibits both procoagulant activity and metastatic capacity of Lewis lung carcinoma cells. *Biochem. Pharmacol.*, **32**, 1689.

DAVIS, R.P., THEOLOGIDES, A. & KENNEDY, B.J. (1969). Comparative study of blood coagulation changes in patients with cancer and with non-malignant diseases. *Ann. Intern. Med.*, **71**, 67.

DOLFINI, E., GHERSA, P., BARBIERI, B. & 3 others (1979). Cytotoxic and cytogenetic effects of nitrogen mustard on EUE cells pretreated with sodium warfarin. *Eur. J. Cancer*, **16**, 77.

DVORAK, H.F., DVORAK, A.M., MANSEAU, E.J., WIBERG, L. & CHURCHILL, W.H. (1979). Fibrin gel investment associated with Line 1 and Line 10 solid tumour growth, angiogenesis and fibroplasia in guinea pigs. *J. Natl Cancer Inst.*, **62**, 1459.

GOETING, N., TROTTER, G.A., COOKE, T., KIRKHAM, N. & TAYLOR, I. (1985). Effect of warfarin on cell kinetics, epithelial morphology and tumour incidence in induced colorectal cancer in the rat. *Gut*, **26**, 807.

HART, I.R. (1980). Cancer invasion and metastasis. *Q. Rev. Biol.*, **55**, 121.

HIGASHI, H. & HEIDELBERGER, C. (1971). Lack of effect of warfarin alone or in combination with 5-fluorouracil on primary and metastatic growth of L1210 leukaemia and adenocarcinoma 755. *Cancer Chemother. Rep.*, **55**, 29.

HILGARD, P., SCHULTE, H., WETZIG, G., SCMITT, G. & SCMIDT, C.G. (1977). Oral anticoagulation in the treatment of a spontaneously metastasising murine tumour (3LL). *Br. J. Cancer*, **35**, 78.

HILGARD, P. & MAAT, B. (1979). Mechanism of lung tumour colony reduction caused by coumarin anticoagulation. *Eur. J. Cancer*, **15**, 183.

McCULLOCH, P.G. & GEORGE, W.D. (1987). Warfarin inhibition of metastasis; the role of anticoagulation. *Br. J. Surg.*, **74**, 879.

McNEIL, N.O. & MORGAN, L.R. JR. (1984). Effects of sodium warfarin and sodium heparin plus anticancer agents on rat C6 glioma cells. *J. Natl Cancer Inst.*, **73**, 169.

MANNUCI, P.M., VAGLINI, M., MANIEZZO, M. & 3 others (1985). Haemostatic alterations are unrelated to stage of tumour in untreated malignant melanoma and breast cancer. *Eur. J. Cancer Clin. Oncol.*, **21**, 681.

NERI, A. & NICOLSON, G.L. (1981). Phenotypic drift of metastatic and cell surface properties of mammary adenocarcinoma cell clones during growth *in vitro*. *Int. J. Cancer*, **28**, 731.

NERI, A., WELCH, D., KAWAGUCHI, T. & NICOLSON, G.L. (1982). The developmental and biological properties of malignant cell sublines and clones of a spontaneously metastasising rat mammary carcinoma. *J. Natl Cancer Inst.*, **68**, 507.

O'REILLY, R.A. (1985). Anticoagulant, antithrombotic and thrombolytic drugs. In *The Pharmacological Basis of Therapeutics*, 7th Edition, Gilman, A.G., Goodman, L.S., Rall, T.W. & Murad, F. (eds). MacMillan: New York.

POSTE, G. & FIDLER, I. (1980). The pathogenesis of cancer metastasis. *Nature*, **283**, 139.

PUCK, T.T. & STEFFEN, J. (1963). Life cycle analysis of mammalian cells. *Biophys. J.*, **3**, 379.

RICKLES, F.R. & EDWARDS, R.L. (1983). Activation of blood coagulation in cancer: Trousseau's syndrome revisited. *Blood*, **62**, 14.

RYAN, J.J., KETCHAM, A.S. & WEXLER, H. (1968). Warfarin treatment of mice bearing autochthonous tumours: Effect on spontaneous metastasis. *Science*, **162**, 1493.

RYAN, J.J., KETCHAM, A.S. & WEXLER, H. (1969). Warfarin therapy as an adjunct to the surgical treatment of malignant tumours in mice. *Cancer Res.*, **29**, 2191.

SEGALOFF, A. (1966). Hormones and breast cancer. *Recent Prog. Horm. Res.*, **22**, 351.

STENFLO, J. & SUTTIE, J.W. (1977). Vitamin K dependent formation of gamma carboxyglutamic acid. *Ann. Rev. Biochem.*, **46**, 157.

SUN, N.C.J., McAFEE, W.M., HUM, G.J. & WEINER, J.M. (1979). Haemostatic abnormalities in malignancy: A prospective study of 108 patients. *Am. J. Clin. Pathol.*, **71**, 10.

SUTTIE, J.W. (1980). Mechanism of action of vitamin K: Synthesis of gamma-carboxy glutamic acid. *CRC Crit. Rev. Biochem.*, **8**, 191.

WEXLER, H. (1966). Accurate identification of experimental pulmonary metastases. *J. Natl Cancer Inst.*, **36**, 641.

WILLIAMSON, R.C.N., LYNDON, P.J. & TUDWAY, A.J.C. (1980). The effects of anticoagulation and ileal resection on the development and spread of experimental intestinal carcinomas. *Br. J. Cancer*, **42**, 85.

ZACHARSKI, L.R., HENDERSON, W.G., RICKLES, F.R. & 10 others (1984). Effect of warfarin anticoagulation on survival in carcinoma of the lung: Head and neck and prostate. *Cancer*, **53**, 2046.